# Empowering the next generation in neuro-oncology: Introduction of the EANO Career Boost Initiative

**DOI:** 10.1093/noajnl/vdae167

**Published:** 2024-10-03

**Authors:** Philipp Lohmann, Johnny Duerinck, Matthijs van der Meulen, Dan Mitrea, Susan Short, Marjolein Geurts

**Affiliations:** Department of Nuclear Medicine, University Hospital RWTH Aachen, Aachen, Germany; Institute of Neuroscience and Medicine (INM-4), Research Center Juelich, Juelich, Germany; C4N - Center for Neurosciences, Vrije Universiteit Brussel, Brussels, Belgium; Department of Neurosurgery, Universitair Ziekenhuis Brussel (UZ Brussels), Brussels, Belgium; Department of Neurology, Medisch Spectrum Twente, Enschede, The Netherlands; Neuroaxis Neurology Clinic, Bucharest, Romania; Leeds Institute of Medical Research at St James’s, The University of Leeds, Leeds, UK; Department of Medical Oncology, Erasmus MC Cancer Institute, University Medical Center, Rotterdam, The Netherlands; Department of Neurology, Brain Tumor Center, Erasmus MC Cancer Institute, University Medical Center, Rotterdam, The Netherlands

**The EANO Career Boost Initiative (CBI) is designed to support neuro-oncologists and researchers in the early stages of their careers in the transition to independence in clinical or research roles. This initiative addresses the challenges of networking, mentoring, and leadership development through dedicated events and a knowledge forum. The CBI provides opportunities for collaboration, access to research knowledge, and leadership development, with a focus on fostering a supportive community for emerging professionals. The initiative aims to address gaps in mentorship, networking, and career development, helping emerging professionals thrive and shape the future of neuro-oncology.**

The medical field is losing many talented professionals early in their careers, including clinicians, researchers, clinician scientists, and other healthcare professionals. This issue spans all specialties and demands urgent attention to ensure the retention of skilled healthcare professionals.

Although several factors such as psychological demands, physical strain of long hours, and intense work environments as well as poor work-life balance apply to all healthcare professionals, those working in neuro-oncology may face even greater challenges. A recent survey from the Society for Neuro-Oncology (SNO) revealed a strikingly high prevalence of burnout among both neuro-oncology clinical and research young investigators, mainly due to overextension.^1^ Patients in neuro-oncology often have a poor prognosis, leading to a higher emotional burden compared to practitioners working in less terminal fields. Moreover, due to the relatively small number of professionals in neuro-oncology compared to other disciplines like radiology or internal medicine, opportunities for adequate mentorship and peer support are hard to find limiting professional networking opportunities especially outside of large, established centers.^2^ The high level of interprofessionalism and interdisciplinarity in combination with the variable orientation of neuro-oncology, particularly in countries where there is no specialization in neuro-oncology, is a major and largely unique challenge to this field.

The rapid evolution of technologies and research paradigms leads to many new opportunities and pathways to understand neuro-oncological disease. Collaboration between groups allows for combining expertise and techniques, as well as bridging the gap between clinical practice and laboratory research. This is crucial to maximize the benefits of advanced techniques and the typically very precious patient samples, and to translate them to a clinical benefit for patients with the help of organizations such as the European Organization for Research and Treatment of Cancer or the National Cancer Institute. Team science and the role of clinician scientists, who promote close collaboration between clinical and laboratory settings, is increasingly important, but there is the challenge of obtaining protected time for scientific research alongside clinical duties.^2,3^

Although several funding schemes specifically designed for early career health care professionals are available, obtaining funding for neuro-oncology research poses a significant challenge. Lack of track-record due to shorter publication records and fewer prior grants make early career scientists less competitive compared to established researchers. There is also the risk-averse nature of funding bodies that prefer to allocate resources to those with proven track records. Hence, there is a decline in the proportion of large research grants going to younger scientists as well as a steady increase in the age at which researchers receive their first funding in biomedical research.^4^

Collaborations with industries will become increasingly necessary, but they come with their own challenges, especially in the early career stages when networks and groups are still developing.

On a more general level, the next generation must address issues of diversity and inclusion within oncology. Underrepresented groups often have less visibility in research and treatment, which impacts the breadth of perspectives and experiences in the field.^5^ Expanding expertise to countries and groups not yet well integrated into the neuro-oncology field presents additional difficulties. Achieving more balanced global progress requires concerted efforts in education, collaboration, and funding.^6^ Gender inequality also persists in neuro-oncology, with women frequently receiving fewer opportunities for leadership positions or research funding.^7,8^

Professional and social networks comprising experienced colleagues and mentors are essential for developing a sustainable, successful, and fulfilling career.^9,10^ However, these networks and the skills required to navigate them are not always readily identifiable or accessible. This can prevent motivated professionals from becoming actively involved in the field—not due to a lack of ability or motivation, but because established structures, committees, resources, and partnerships can be challenging to access without support.

**Figure 1.**
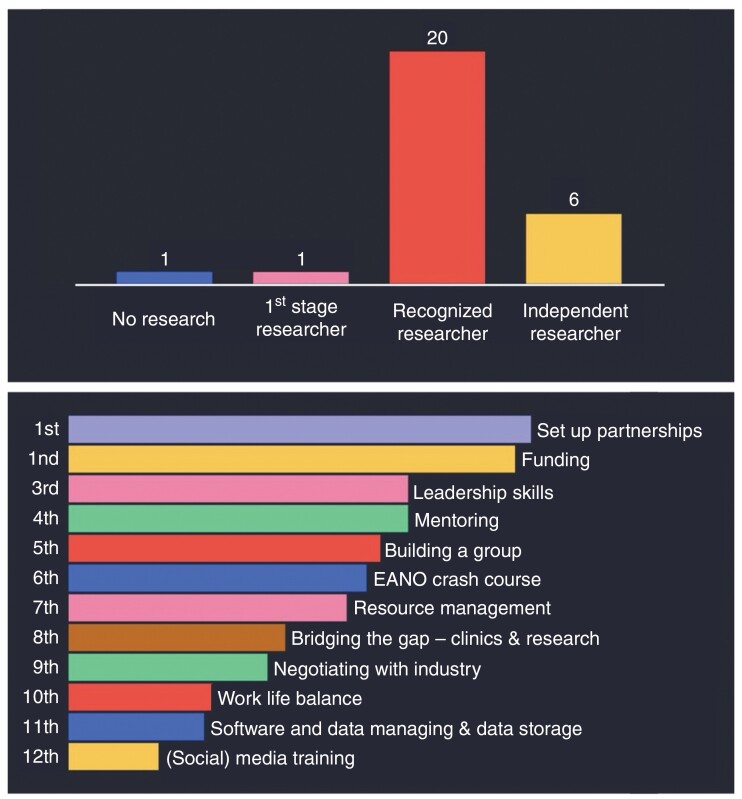
Results from survey conducted at 2023 EANO Annual Meeting. Top panel: Most participants considered themselves as recognized researchers, i.e. PhD holders or equivalent who are not yet fully independent. Bottom panel: Participants most strongly expressed their need for setting up scientific partnerships, get access to funding, and gaining knowledge regarding leadership skills and mentoring.

The challenges within this group of people who are working toward clinical or scientific independence became evident in a survey that was conducted at an open and informal meeting at the EANO Annual Meeting in Rotterdam in 2023, hosting 44 people. Most participants considered themselves as recognized researchers, i.e. PhD holders or equivalent who are not yet fully independent (Figure 1). The participants most strongly expressed their need for networking (e.g. better support to set up scientific partnerships that might result in joint funding applications), and gaining knowledge regarding leadership skills (e.g. how to build a group, learning more about the structure and ways to be actively engaged in EANO, manage resources, bridging the gap between clinics and research, negotiating with industry, work-life-balance, software and data managing, and (social) media training; Figure 1). This first informal meeting highlighted the need for targeted support for this specific group of people.

We think these challenges can and should be addressed on an associational level such as the Society of Neuro-Oncology (SNO) or the European Association of Neuro-Oncology (EANO), in line with their missions.

SNO features, besides the Career Development Events that are well established at the SNO Annual Meeting, SNO a recently launched Future Leaders Development Program for young physician-scientists. In the program, an experienced instructor together with a senior SNO leader provides practical, hands-on education in different topic areas such as emotional intelligence, critical thinking, finding your niche, and the economics of leading a department. Although this initiative is important and highly appreciated, the application process is very competitive and only available for a small number of people from North America.

In Europe, the European Society for Medical Oncology places a strong emphasis on career development through various initiatives such as Fellowships, a Mentoring program, a specialized Young Oncologist track at their annual scientific meeting, and Y04Y0 virtual session meetings, among others. However, these resources are primarily geared toward medical oncologists and remain largely inaccessible to the multidisciplinary field of neuro-oncologists.

Within EANO, the Youngsters Committee has become highly effective in supporting neuro-oncologists in the very early stages of their careers. However, the age restriction (<35 years) leaves a gap for individuals who are in the process of building their independent clinical and/or research career in neuro-oncology

To address the needs of European aspiring physicians and researchers working toward independence in neuro-oncology, the EANO Career Boost Initiative (CBI) is launched. The initiative aims to support emerging physicians and researchers in the field of neuro-oncology on their path to scientific and/or clinical independence, recognition and active engagement by supporting a gateway to vital connections, collaborative opportunities, and leadership growth. The CBI aims to address the multifaceted aspects of cultivating a successful career in clinical or experimental neuro-oncology by organizing networking events as well as providing dedicated CBI session addressing, leadership skills, and more.

A key element of the CBI is the Knowledge Forum, an innovative platform designed to facilitate informal and open exchanges, as well as to provide a dynamic environment for networking and collaboration. The Knowledge Forum will be introduced and launched at the 2024 EANO Annual Meeting in Glasgow. This platform aims to connect aspiring clinicians and researchers with more established research groups and institutions, granting access to a wide range of methods and expertise that would otherwise be inaccessible, thereby initiating multinational and multicentric collaborations. By sharing knowledge and granting access to specialized methods, CBI fosters project-driven collaborations that facilitate funding access and enhance funding success rates. These collaborations lead to the development of networks that positively impact careers beyond specific projects and promote integration within the community.

The EANO CBI represents a significant commitment to the future of neuro-oncology. By investing in the potential of aspiring talents and providing a comprehensive support framework, EANO aims to contribute to the personal and collective development of these professionals and the field at large. This initiative promises to create a dynamic and supportive environment where the next generation of neuro-oncologists can thrive, innovate, and lead the field into a promising future. Bringing together the SNO and EANO initiatives would be the next step to jointly develop the next generation of international leaders in the field of neuro-oncology.
